# Transcriptional regulators *TRIM28*, *SETDB1*, and *TP53* are aberrantly expressed in porcine embryos produced by in vitro fertilization in comparison to in vivo- and somatic-cell nuclear transfer-derived embryos

**DOI:** 10.1002/mrd.22324

**Published:** 2014-04-16

**Authors:** Jennifer Hamm, Kim Tessanne, Clifton N Murphy, Randall S Prather

**Affiliations:** 1 Division of Animal Sciences, University of MissouriColumbia, Missouri

## Abstract

In vitro embryo production is important for research in animal reproduction, embryo transfer, transgenics, and cloning. Yet, in vitro-fertilized (IVF) embryos are generally developmentally delayed and are inferior to in vivo-derived (IVV) embryos; this discrepancy is likely a result of aberrant gene expression. Transcription of three genes implicated to be important in normal preimplantation embryo development, *TRIM28*, *SETDB1*, and *TP53*, was determined by quanitative PCR in IVF, somatic-cell nuclear transfer (SCNT), parthenogenetic, and IVV porcine oocytes and embryos. There was no difference in *TRIM28* or *SETDB1* abundance between oocytes matured in vitro versus in vivo (*P* > 0.05), whereas *TP53* levels were higher in in vitro-matured oocytes. *TRIM28* increased from metaphase-II oocytes to the 4-cell and blastocyst stages in IVF embryos, whereas IVV embryos showed a reduction in *TRIM28* abundance from maturation throughout development. The relative abundance of *TP53* increased by the blastocyst stage in all treatment groups, but was higher in IVF embryos compared to IVV and SCNT embryos. In contrast, *SETDB1* transcript levels decreased from the 2-cell to blastocyst stage in all treatments. For each gene analyzed, SCNT embryos of both hard-to-clone and easy-to-clone cell lines were more comparable to IVV than IVF embryos. Knockdown of *TRIM28* also had no effect on blastocyst development or expression of *SETDB1* or *TP53*. Thus, *TRIM28*, *SETDB1*, and *TP53* are dynamically expressed in porcine oocytes and embryos. Furthermore, *TRIM28* and *TP53* abundances in IVV and SCNT embryos are similar, but different from quantities in IVF embryos. *Mol. Reprod. Dev. 81: 552–556, 2014. © 2014 The Authors*. Published by Wiley Periodicals, Inc.

## INTRODUCTION

Oocyte maturation and early embryonic development are sensitive to perturbations in their regulation, which appear as altered embryo growth and quality. In vitro-derived embryos generally exhibit delayed development and are inferior to in vivo-derived (IVV) embryos. Even with improved in vitro embryo culture conditions, the artificial environment remains less than optimal. Preimplantation embryos of agricultural animals are particularly susceptible, as embryo manipulation or suboptimal culture environments generally result in abnormal placental, fetal, and postnatal development (McEvoy et al., 1998; Sinclair et al., 1999; Peterson et al., 2000; Van Wagtendonk-de Leeuw et al., 2000; McEvoy et al., 2001). The stresses inflicted on in vitro-derived embryos can lead to poor embryo quality and a change in global gene expression (Rinaudo and Schultz, 2004; Whitworth et al., 2005; Rinaudo et al., 2006).

Pigs generated by nuclear transfer of genetically engineered cells play an important role in the generation of biomedical models for the research of human and animal disease, pharmaceutical development, and improvements in animal agriculture (Prather et al., 2008; Prather, 2013). Yet, nuclear transfer is still inefficient for producing live births in most species: less than 1% in pigs (Colman, 2000), 5–8% in sheep, and 10–15% in cattle (Cibelli et al., 1998; Wells et al., 2003).

TRIM28, or TIF1β, is a nuclear protein thought to function as a co-repressor of the zinc-finger proteins associated with the Kruppel-associated box (KRAB) domain (Friedman et al., 1996). In mammals, KRAB functions as a transcriptional repression domain, exhibiting the capacity for long-range transcriptional repression across much of the genome (Margolin et al., 1994; Witzgall et al., 1994; Pengue et al., 1995; Vissing et al., 1995; Cammas et al., 2000). TRIM28 and multiple KRAB domains repress transcriptional activity by directly binding to DNA (Friedman et al., 1996; Kim et al., 1996; Moosman et al., 1996; Agata et al., 1999; Nielsen et al., 1999; Cammas et al., 2000). In the mouse, TRIM28 is essential for post-implantation development, normal gene expression, and epigenetic stability during the zygotic transition (Cammas et al., 2000; Whitelaw et al., 2010; Messerschmidt et al., 2012). TRIM28 can also promote ubiquitination and degradation of TP53, a tumor suppressor.

TP53 helps protect genome integrity (Livingstone et al., 1992; Yin et al., 1992) and maintain general cell homeostasis. In the presence of chromosomal mutations or damage, TP53 acts through multiple mechanisms—such as apoptosis, cell cycle arrest, or cellular senescence (Vogelstein et al., 2000; Brooks and Gu, 2006) to halt the propagation of any mutated DNA. In the absence of stress, cells closely regulate TP53 levels, keeping TP53 inactive or its protein levels low (Brooks and Gu, 2006). TP53 is also believed to respond to stress caused by suboptimal growth conditions (Donehower and Bradley, 1993), including heat stress, starvation, and hypoxia (Zhan et al., 1993; Graeber et al., 1994). Current models suggest that TP53-dependent apoptosis is regulated through activity of TRIM28, HDAC1 (histone deacetylase I), and ZNF420 (ATM and TP53-associated KZNF protein (APAK)). In the absence of DNA damage or internal stress, ZNF420 binds to TP53 (Yuan et al., 2012) and TRIM28, which binds to HDAC1; this complex drives the deacetylation of TP53, thus repressing proapoptotic gene expression (Tian et al., 2009). In stressed cells, however, apoptosis is triggered by phosphorylation of ZNF420 and TRIM28 (Ziv et al., 2006), causing ZNF420 to detach from TRIM28 and relocate into the nucleolus (Yuan et al., 2012). Phosphorylated TRIM28 no longer facilitates the deacetylation of TP53 by HDAC1, thereby allowing acetylated TP53 to activate proapoptotic genes.

SETDB1 (SET domain bifurcated 1, or ESET) is a histone methyltransferase involved in epigenetic transcriptional repression, mostly in euchromatic genes. SETDB1 trimethylates lysine-9 of histone 3 (H3K9) through a direct interaction with TRIM28 (Schultz et al., 2002). Knockout of *Setdb1* in mice results in embryos that die shortly after implantation due to improper development of the primitive ectoderm (Dodge et al., 2004), but the blastocysts have seemingly normal global di- and tri-methylation of H3K9. Thus, although maternal SETDB1 is essential for peri-implantation and development of primitive ectoderm, the inner cell mass, and embryonic stem cells, it likely has no role in global histone methylation in preimplantation development.

As previous research has indicated that blastocyst formation is an inadequate indicator of embryo normality (Messerschmidt et al., 2012), additional metrics for poor embryo quality need to be identified to improve embryo production for research and for the generation of biomedical models. Therefore, a goal of this project was to characterize *TRIM28*, *SETDB1*, and *TP53* expression in in vitro fertilized (IVF), somatic-cell nuclear transfer (SCNT), parthenogenetic, and IVV porcine oocytes and embryos throughout porcine oocyte maturation and preimplantation development. Although previous studies (Cammas et al., 2000; Whitelaw et al., 2010; Messerschmidt et al., 2012) have examined morphological and epigenetic impacts of *Trim28* on embryo development in knockout mice, it has not yet been investigated in other mammals. Additionally, in vitro-cultured embryos have not been examined. Thus, we designed a series of experiments to further investigate differences between porcine embryos produced in vitro versus in vivo, particularly to gain insight into the embryonic mechanisms underlying suboptimal in vitro embryo production and live-birth inefficiency from SCNT protocols.

## RESULTS

### TRIM28 Abundance Is Higher in Porcine IVF Embryos

**Figure 1:**
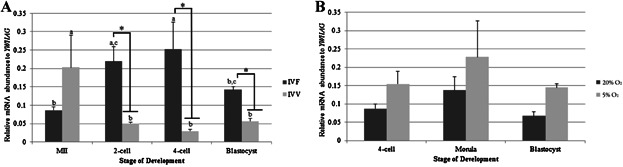
qPCR for *TRIM28*. A: *TRIM28* increases in IVF embryos at the 2-cell and 4-cell stages compared to the MII oocyte. IVV embryos have decreased *TRIM28* abundance from the 2-cell stage through the blastocyst stage relative to the MII oocyte. *TRIM28* is higher in IVF embryos versus IVV embryos at the 2-cell, 4-cell, and blastocyst stages. Relative mRNA abundance was normalized to *YWHAG*. B: *TRIM28* abundance was sensitive to oxygen partial pressure, with an overall increase in 5% O_2_ compared to 20% O_2_ embryos (*P* = 0.03). No stage-effect was observed. Relative mRNA abundance was normalized to *YWHAG*. Letters (a, b, etc.) denote differences between stages *within a method* of production (*P* < 0.009). *Denotes differences *within a stage* (*P* < 0.007). Error bars represent standard error.

Quantitative reverse-transcriptase PCR (qPCR) was first used to profile *TRIM28* mRNA abundance in metaphase-II (MII) oocytes and embryos created either in vitro or in vivo. The method of oocyte maturation had no significant effect on *TRIM28* transcript levels (Fig. 1A). The abundance of *TRIM28* in IVV embryos dropped at the 2-cell stage and stayed low until the blastocyst stage. In contrast, IVF embryos contained more *TRIM28* at the 2-cell and 4-cell stages compared to the MII oocytes and blastocysts. This pattern resulted in greater overall quantities of *TRIM28* in IVF embryos than in IVV embryos.

### Oxygen Partial Pressure Affects TRIM28 Abundance in IVF Embryos

qPCR was also used to quantify the message level of *TRIM28* in IVF 4-cell, morula, and Day-6 blastocyst-stage embryos that were cultured in 20% oxygen (5% CO_2_ in atmospheric air) or 5% oxygen (5% CO_2_, 5% O_2_, and 90% N_2_) to assess the effects of oxygen partial pressure on *TRIM28* expression. Relative transcript abundance was analyzed in two manners: by stage of development versus oxygen partial pressure and by overall interaction of 20% versus 5% O_2_ treatment. When relative *TRIM28* abundance was compared between oxygen environments at a specific stage of development (i.e., at the 4-cell stage, 20% vs. 5% O_2_), there was no observed developmental-stage effect. A general effect of oxygen partial pressure was detected, however, with 5% O_2_ levels resulting in more *TRIM28* mRNA (*P* = 0.03) (Fig. 1B).

### The Increase of TRIM28 Abundance in IVF Embryos is not Transcription- or Polyadenylation-Dependent After Fertilization

**Figure 2:**
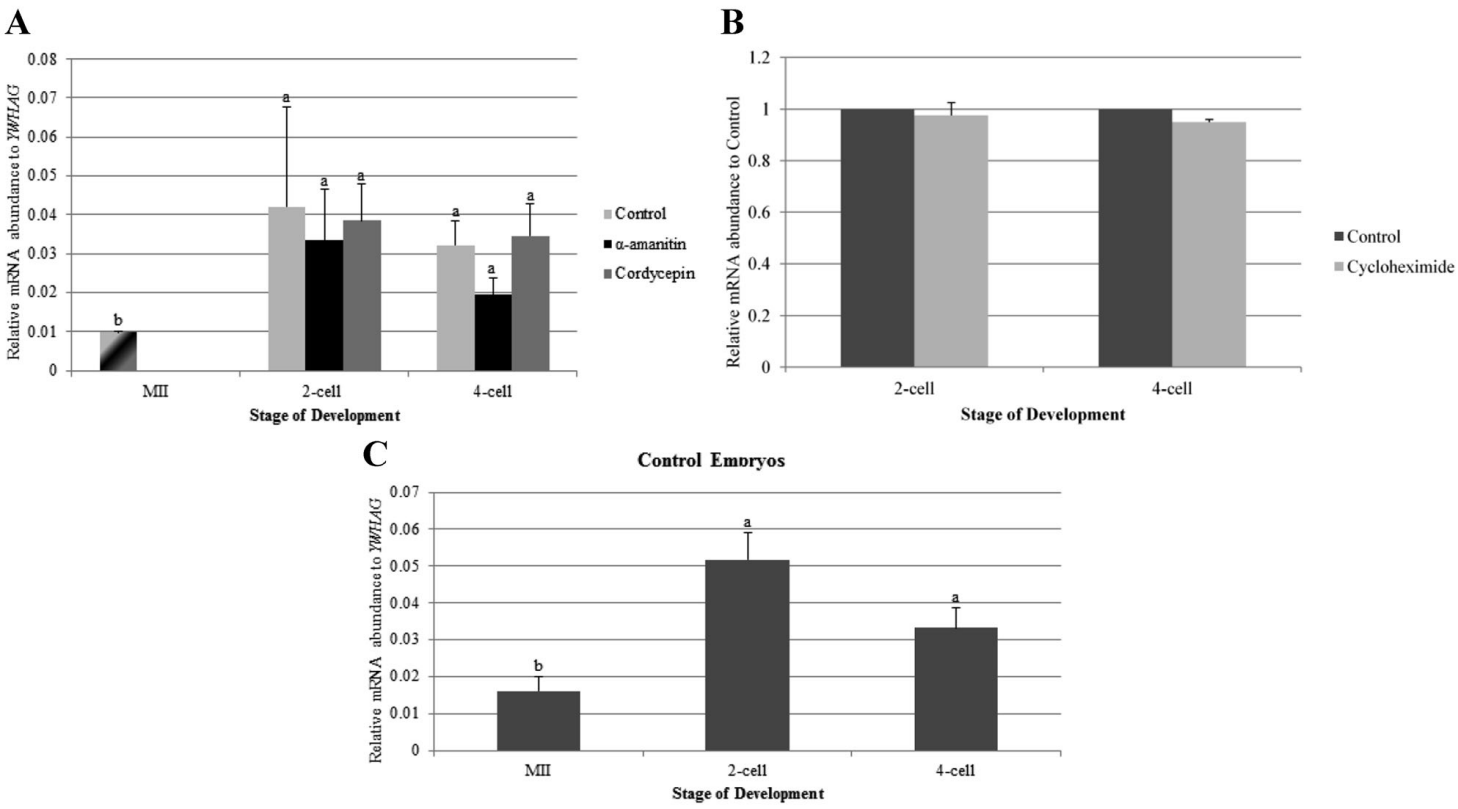
qPCR for *TRIM28* in IVF embryos cultured in α-amanitin, cordycepin, or cycloheximide *after* fertilization. A: Inhibition of transcription and polyadenylation *after* fertilization with α-amanitin and cordycepin, respectively, had no effect on *TRIM28* expression, which increased at the 2-cell and 4-cell stages for all treatment groups. MII oocytes were used as a control for all stages. Relative mRNA abundance was normalized to *YWHAG*. B: *TRIM28* abundance was similar at the 2-cell and 4-cell stages for control and cycloheximide-treated embryos. Relative mRNA abundance was normalized to Control counterparts. C: Control embryos demonstrated the previously described increase of *TRIM28* from MII to the 2-cell and 4-cell stages. Relative mRNA abundance was normalized to *YWHAG*. Letters (a, b) denote differences *between stages* (*P* < 0.04). Error bars represent standard error.

A significant increase in the abundance of *TRIM28* occurs at the 2-cell and 4-cell stages of IVF embryos compared to MII oocytes. We first wanted to determine if this increase could be due to polyadenylation of pre-existing message or to new transcription. Alpha-amanitin was used to inhibit RNA polymerase II (Schoenbeck et al., 1992), thus preventing transcription, while treatment with cordycepin was used to prevent the elongation of poly-adenine tails (Dobbs et al., 2010). We drug-treated embryos after fertilization (4–6 hr after the addition of sperm) until the time of collection. No change in the accumulation of *TRIM28* transcripts was observed with 22 µM α-amanitin or 40 µM cordycepin (Fig. 2A). Thus, the increase in *TRIM28* abundance in cleavage-stage embryos is not dependent upon transcription or polyadenylation.

### Inhibition of Protein Synthesis Has No effect On TRIM28 mRNA Abundance in IVF Embryos After Fertilization

As the increase of TRIM28 transcript abundance from the MII oocyte to the 2-cell and 4-cell stages is independent of transcription and polyadenylation *after* fertilization, protein synthesis was inhibited in IVF embryos using 35 µM cycloheximide. Cycloheximide-treated embryos did not cleave, so treated embryos were collected at the same time point that control embryos reached the 2- or 4-cell stage. The relative abundance of *YWHAG* (tyrosine 3-monooxygenase/tryptophan 5-monooxygenase activation protein gamma) mRNA was significantly different between cycloheximide-treated and media-alone control embryos at the 2-cell and 4-cell stages, thus making *YWHAG* an unsuitable normalizer for qPCR. Instead, values for *TRIM28* abundance were normalized to the abundance of *TRIM28* in untreated control embryos for that corresponding biological replicate. Relative *TRIM28* mRNA abundance did not differ between control and cycloheximide-treated embryos at the 2-cell or 4-cell stages (Fig. 2B). Furthermore, the same significant increase in *TRIM28* transcript abundance was observed in the MII oocyte and in the IVF 2-cell and 4-cell control embryos (Fig. 2C).

### Increase of TRIM28 mRNA Abundance in IVF Embryos May Be Translation-Dependent During Fertilization

Since the relative increase of *TRIM28* mRNA abundance at the 2-cell and 4-cell stages were not a result of transcription, polyadenylation, or translation *after* fertilization, we looked to effects of inhibiting transcription, polyadenylation, and translation *during* fertilization. Inhibitors were therefore added at the same time as sperm, until the 2-cell stage (or the equivalent time for the non-cleaving, cycloheximide-treated embryos). Oocytes were treated as described above, then *TRIM28* levels were quantified in MII oocytes and 2-cell stage embryos in unamplified samples (thus the ratios differ from those in Figs. 1 and 2).

**Figure 3:**
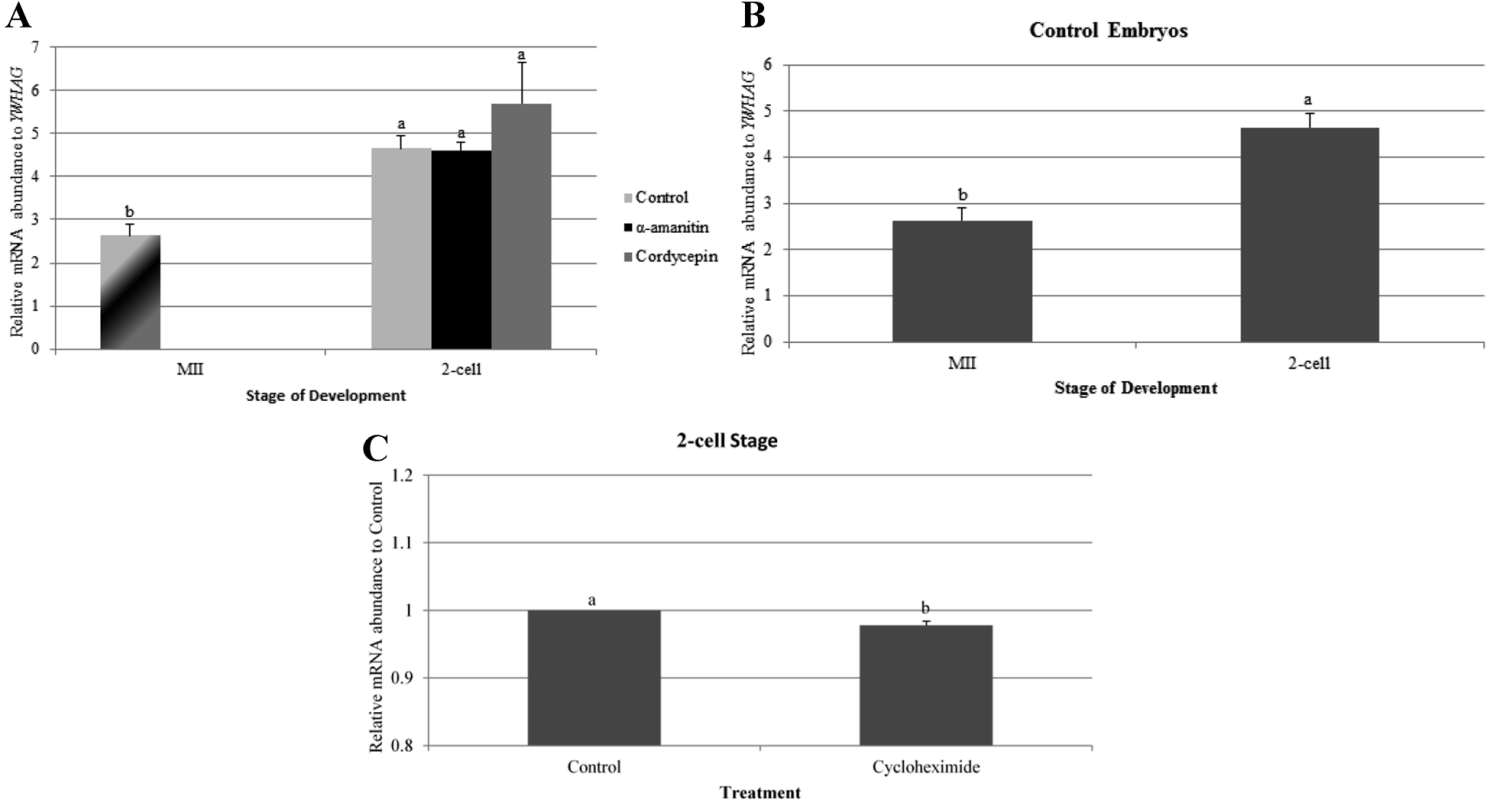
qPCR for *TRIM28* in IVF embryos cultured in α-amanitin, cordycepin, or cycloheximide *during and after* fertilization. A: Inhibition of transcription and polyadenylation *during and after* fertilization had no impact on *TRIM28* abundance, as it increased from the MII oocyte to the 2-cell stage for all treatment groups. There was no difference of *TRIM28* expression in treated embryos at the 2-cell stage. MII oocytes were used as a control for all stages. Relative mRNA abundance was normalized to *YWHAG*. B: Control embryos demonstrated the increase of *TRIM28* from MII to the 2-cell stage. Relative mRNA abundance was normalized to *YWHAG*. C: Cycloheximide-treated, 2-cell stage embryos had a decrease in *TRIM28* abundance compared to control embryos. Relative mRNA abundance was normalized to Control counterparts. Letters (a, b) denote differences between stages *within a method* of production (*P* < 0.03). These were unamplified samples, and thus the ratio is different from the previous graphs. Error bars represent standard error.

An increase in *TRIM28* abundance at the 2-cell stage still occurred, despite the inhibition of translation and polyadenylation during fertilization using α-amanitin or cordycepin, respectively (Fig. 3A). Control embryos for the protein synthesis inhibition experiment also showed an increase in *TRIM28* levels from the MII oocyte to the 2-cell stage (Fig. 3B). Cycloheximide-treated 2-cell embryos, however, had less *TRIM28* mRNA (again, not normalized to *YWHAG*) when compared to control embryos (Fig. 3C). Thus, the increase of *TRIM28* in IVF embryos at the 2-cell stage seems to be affected by inhibition of protein synthesis *during* fertilization.

### TRIM28 Abundance is Similar in Porcine SCNT and Parthenogenetic Embryos

**Figure 4:**
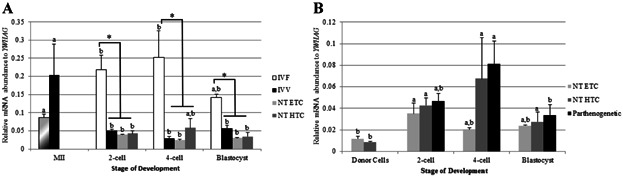
qPCR for *TRIM28* in in vitro- and in vivo-derived embryos. A: SCNT embryos were more similar to IVV embryos, with a decreased *TRIM28* abundance from the 2-cell stage through the blastocyst stage relative to the MII oocyte. IVF embryos had more *TRIM28* in 2-cell, 4-cell, and blastocyst-stage embryos in relation to IVV and SCNT embryos. MII oocytes were used as a control for all stages in IVF and SCNT embryos. B: Parthenogenetic and NT embryos displayed a similar trend of *TRIM28* expression. Donor cells had relatively low *TRIM28* abundance compared to the 2-cell and 4-cell stages. Relative mRNA abundance was normalized to *YWHAG*. Letters (a, b, etc.) denote differences *within a method* of production (*P* < 0.034). Asterisks (*) denote differences *within a stage* (*P* < 0.038). Error bars represent standard error.

qPCR was also used to invesitgate if SCNT embryos derived from hard-to-clone (HTC) donor cell lines have inadequate *TRIM28* transcript abundance in comparison to SCNT embryos from easy-to-clone (ETC) donor cell lines (see Materials and Methods section for a description of these two classes of cells). There was no difference in *TRIM28* abundance at the 2-cell, 4-cell, or blastocyst stages between SCNT-HTC and SCNT-ETC embryos (Fig. 4A). When both SCNT treatments were compared to IVF and IVV embryos, *TRIM28* levels in SCNT embryos were closer to IVV embryos; IVF embryos had more *TRIM28* transcripts at the 2-cell, 4-cell, and blastocyst stages than either IVV or SCNT embryos. Both sets of SCNT embryos had the same pattern of expression as IVV oocytes and embryos, with a significant decrease in *TRIM28* levels from MII oocytes to 2-cell, 4-cell, and blastocyst stages.

The abundance of *TRIM28* in the SCNT-HTC embryos was higher than in their donor cells, but there was no difference in transcript levels between the SCNT-ETC embryos and their donor cells at the 4-cell and blastocyst stages (Fig. 4B). Similarly, *TRIM28* abundance in parthenogenetic embryos was no different than SCNT embryos. Overall, *TRIM28* expression in SCNT and parthenogenetic embryos is relatively low, and remains low or significantly decreases throughout development.

### SETDB1 Abundance Decreases Throughout Development in Porcine Embryos

**Figure 5:**
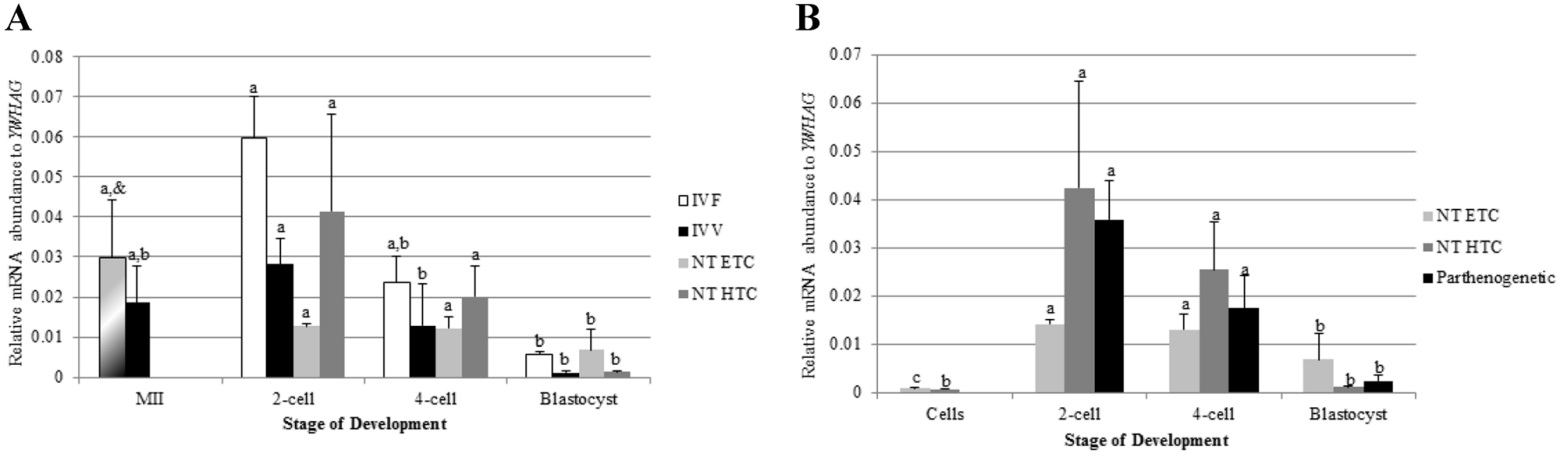
qPCR for *SETDB1* in in vitro- and in vivo-derived embryos. A: *SETDB1* abundance decreases from MII oocytes to the blastocyst stage in all treatments. Similar *SETDB1* expression was observed in all stages for all treatments. MII oocytes were used as a control for all stages in IVF and SCNT embryos. B: Donor cells have a low *SETDB1* abundance, and SCNT and parthenogenetic embryos decrease in abundance during development, returning to a level similar to that of the donor cells. Relative mRNA abundance was normalized to *YWHAG*. Letters (a, b, etc.) denote differences *within a method* of production (*P* < 0.002). Error bars represent standard error.

qPCR was used to quantify *SETDB1* mRNA abundance throughout embryonic development in IVF embryos to investigate if aberrant *SETDB1* levels could result from suboptimal embryonic development conditions. In vitro versus in vivo maturation had no impact on *SETDB1* expression in MII oocyte or on embryogenesis, with abundance significantly decreasing from early stages to the blastocyst stage for all treatments (Fig. 5A).

Donor cells had less *SETDB1* mRNA relative to the early embryos; levels in these somatic lines were similar to those at the blastocyst stage (Fig. 5B). Parthenogenetic embryos have the same trend of expression across development as both SCNT groups. The transcript profile in early porcine oocytes and embryos is also similar to the SETDB1 protein expression seen in mouse oocytes and embryos, where maternal SETDB1 in mouse embryos decreases throughout development, until zygotic expression begins at the late-blastocyst stage (Cho et al., 2012).

### TP53 Abundance in Porcine Preimplantation Oocytes and Embryos

**Figure 6:**
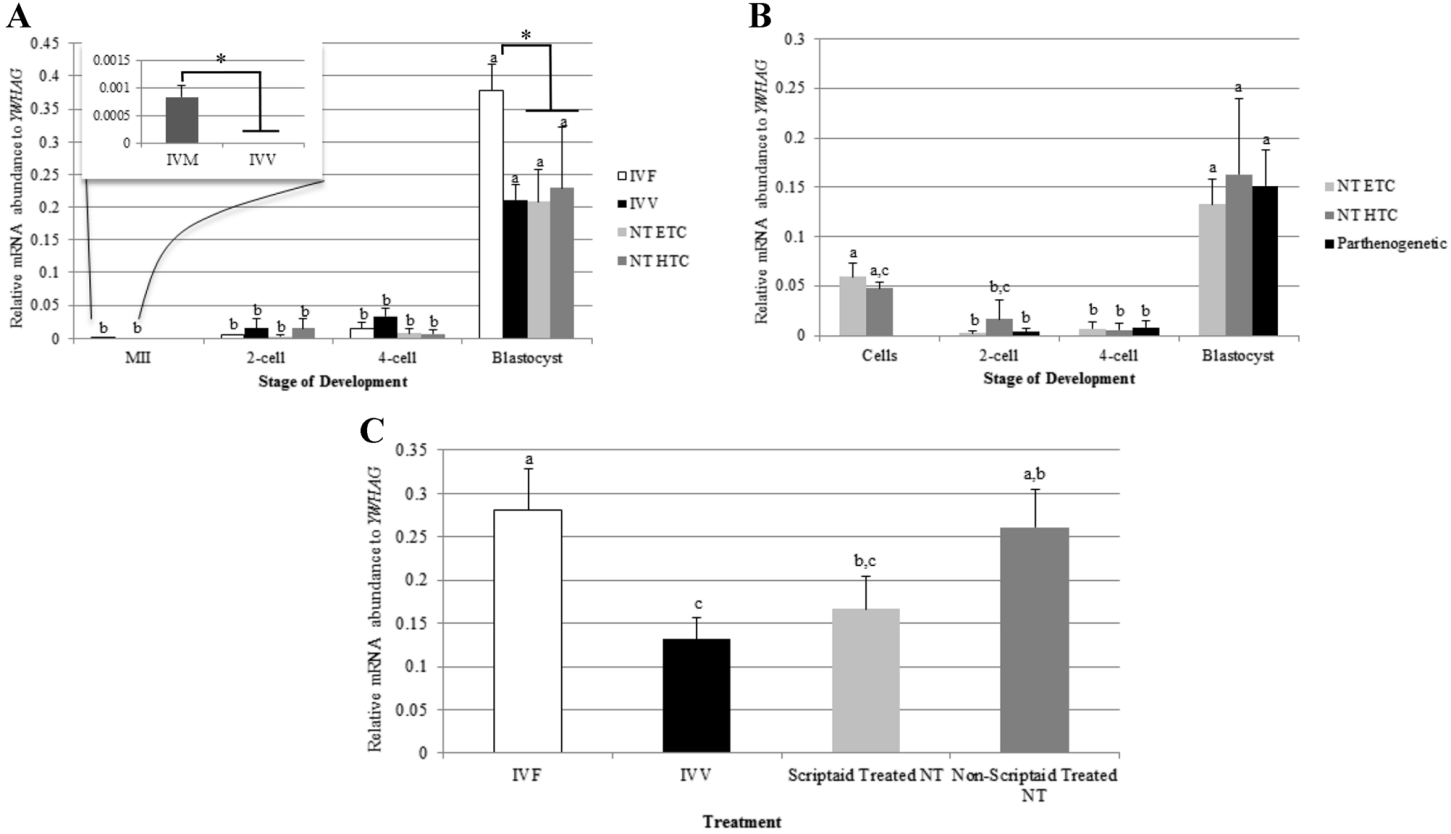
qPCR for *TP53*. A: MII oocytes matured in vitro have an increased abundance of *TP53* in comparison to in vivo-matured oocytes (inset). *TP53* abundance remains low throughout development, and increases at the blastocyst stage. IVF embryos have higher amounts of *TP53* than IVV and SCNT blastocysts. MII oocytes were used as a control for all stages in IVF and SCNT embryos. B: SCNT and parthenogenetic embryos have an increased *TP53* abundance at the blastocyst stages compared to the 2- and 4-cell stages. Donor cells have similar *TP53* expression as in the blastocyst stage. C: *TP53* was higher in IVF and non-Scriptaid-treated SCNT blastocysts in comparison to IVV embryos. Relative mRNA abundance was normalized to *YWHAG*. Letters (a, b, etc.) denote differences (*P* < 0.048) *within a method* of production for panels (a) and (b), and between methods for panel (c). Asterisks (*) denote differences *within a stage* (*P* < 0.009). Error bars represent standard error.

*TP53* levels were characterized by qPCR in IVF, IVV, SCNT, and parthenogenetic preimplantation oocytes and embryos to determine if abnormal *TRIM28* expression in IVF embryos affects *TP53* expression. While the levels are generally very low, *TP53* abundance is higher in in vitro-matured MII oocytes compared to in vivo-matured oocytes (Fig. 6A inset). At the 2-cell and 4-cell stages, there was no difference seen in *TP53* transcript abundance between IVF, IVV, and SCNT embryos. At the blastocyst stage, IVF embryos had more *TP53* mRNA than IVV or SCNT embryos, which coincides with a greater abundance of *TRIM28* at the blastocyst stage in IVF embryos. Across preimplantation development, *TP53* abundance remains exceptionally low, but significantly increases at the blastocyst stage for all treatment groups.

Donor cells for both HTC and ETC cell lines have similar *TP53* levels as the blastocyst stage, which were significantly higher than the 2-cell and 4-cell stages (Fig. 6B). Parthenogenetic embryos demonstrate the same pattern of *TP53* expression, with a small quantity at the 2-cell and 4-cell stages and a significant increase at the blastocyst stage.

### Scriptaid-Treated SCNT Embryos Have Decreased TP53 Abundance at the Blastocyst Stage

To determine if the use of Scriptaid for 14–16 hr after activation was affecting *TP53* abundance at the blastocyst stage, qPCR was performed on Scriptaid-treated and non-treated SCNT embryos from a different ETC cell line. Non-treated SCNT blastocysts had higher *TP53* expression than IVV embryos, with no difference in abundance compared to IVF blastocysts (Fig. 6C). Thus, treating SCNT embryos with Scriptaid decreases *TP53* abundance at the blastocyst stage, making them more similar to IVV embryos.

### TRIM28 Knockdown in Oocytes Has No Effect on Blastocyst Development

**Figure 7:**
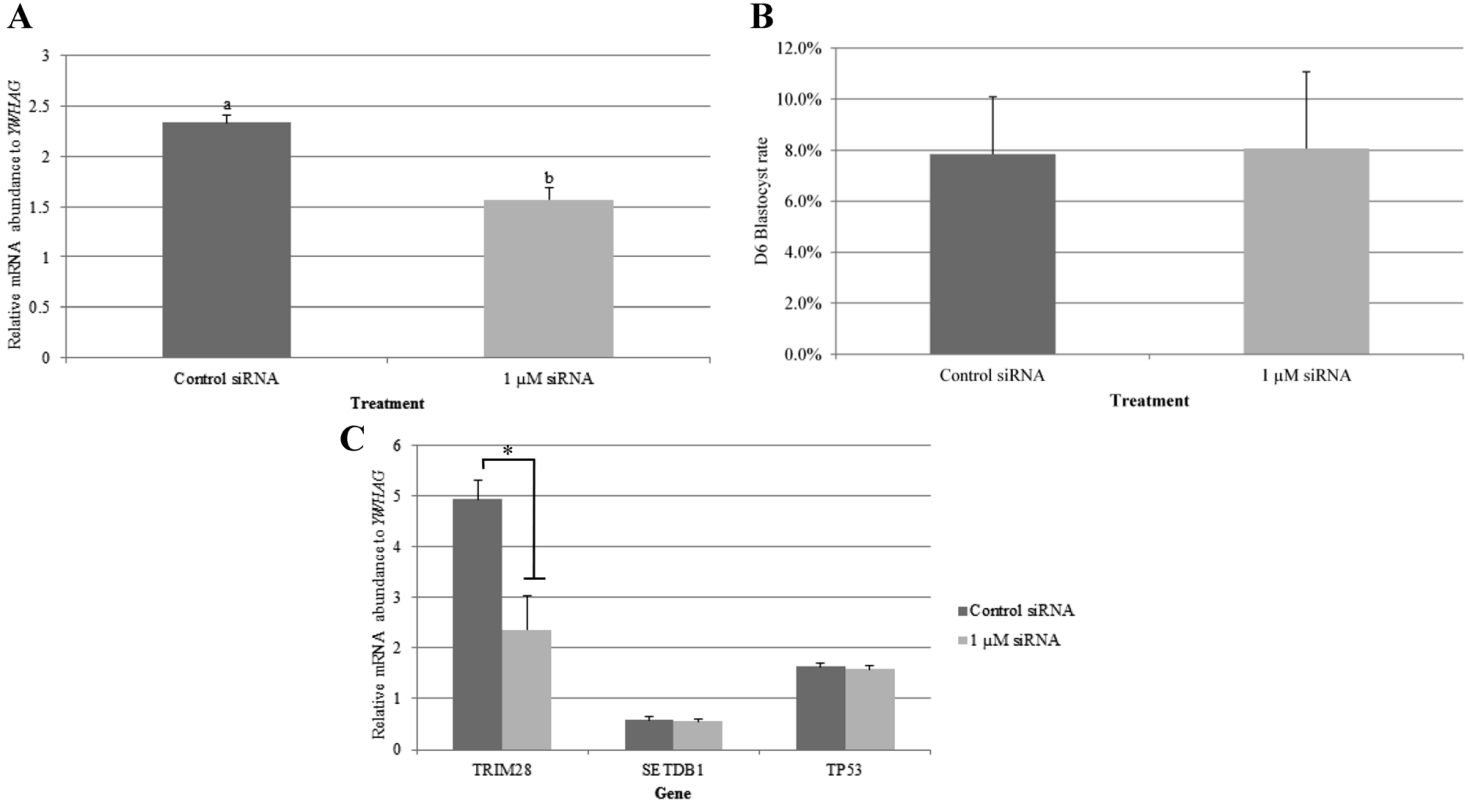
qPCR for *TRIM28* mRNA abundance in MII oocytes injected with *TRIM28* siRNA and *GFP* mRNA. A: Successful knockdown of *TRIM28* was achieved in 1 µM siRNA-injected embryos, with a 30% decrease in abundance (*P* = 0.008). B: Day-6 blastocyst rates were not affected by *TRIM28* knockdown (*P* = 0.58). C: Knockdown of *TRIM28* in MII oocytes did not affect *TP53* or *SETDB1* abundance at the blastocyst stage. A decrease in *TRIM28* at the blastocyst stage was observed in siRNA-injected embryos (*P = 0.04). Relative mRNA abundance was normalized to *YWHAG*. Note that these were unamplified samples and thus the ratios are different from previous graphs. Error bars represent standard error.

The *TRIM28* coding sequence was determined and compared to the human, bovine, mouse, and rat coding sequences (Table1). Porcine *TRIM28* shared 97% amino acid homology to the human and bovine sequences. Small-interfering RNA (siRNA) knockdown of *TRIM28* in the porcine oocyte was performed to determine if *TRIM28* abundance affects *TP53* or *SETDB1*. (Again, it should be noted that these were unamplified samples, thus the ratios are different from those in previous figures). In preliminary experiments, 1, 10, or 50 µM siRNA was injected; 1 µM resulted in the greatest knockdown (2 replicates; data not shown) while another experiment showed that 1 µM resulted in a similar knockdown as 0.1 and 0.01 µM (data not shown). Microinjection of 1 µM siRNA resulted in 33% knockdown of *TRIM28* 10 hr later in the MII oocyte (Fig. 7A), and had no impact on Day-6 blastocyst rates (Fig. 7B). Additionally, knockdown of *TRIM28* had no impact on *SETDB1* or *TP53* expression at the blastocyst stage (Fig. 7C), despite showing a 50% decrease in its own transcript levels at the blastocyst stage in siRNA-injected embryos.

**Table 1:** Homology of *TRIM28* Porcine Coding Sequence

	Nucleotide	Amino acid
Length of porcine coding sequence	2,666	836
Identity against human	88%	97%
Identity against bovine	91%	97%
Identity against mouse	84%	95%
Identity against rat	83%	95%

## DISCUSSION

*TRIM28* abundance during preimplantation embryo development is known to be crucial for post-implantation embryo survival and normal fetal development (Cammas et al., 2000; Messerschmidt et al., 2012). We initially hypothesized that *TRIM28* expression was significantly reduced in in vitro-derived embryos, possibly leading to aberrant methylation in specific imprinted genes. In addition to profiling *TRIM28* expression, we investigated *SETDB1* abundance in preimplantation embryos. *SETDB1*, a histone methyltransferase, cooperates with *TRIM28* to maintain trimethylation of H3K9, and plays an important role in post-implantation embryo survival in mice (Dodge et al., 2004).

This study established that the type of oocyte maturation has no effect on *TRIM28* transcript abundance at the time of the first-polar-body extrusion. Culturing embryos in vitro affects *TRIM28* expression in later embryonic development at the 2-cell and 4-cell stage, however. In addition to a significant increase in *TRIM28* expression at the 2-cell, 4-cell, and blastocyst stages, IVF embryos also exhibit an altered pattern of expression throughout development compared to IVV embryos. IVV embryos reduce their *TRIM28* transcript levels from the MII oocyte to the 2-cell, 4-cell, and blastocyst stages, whereas in vitro-derived embryos significantly increase *TRIM28* expression from the MII stage to the 2-cell and 4-cell-embryo stages. The cause for this increase during the early-cleavage stages is unknown: The elevated *TRIM28* expression appears to be independent of polyadenylation and transcription during or after fertilization, but is sensitive to protein synthesis (translation) inhibitors *during* but *not after* fertilization. Since there is little, if any, transcription between germinal vesicle breakdown and the 4-cell stage in the pig, the observed increase in *TRIM28* mRNA abundance is perplexing. Explanations for this observation might include a change in the abundance of housekeeping genes, although our threshold values were not different for *YWHAG*. A technical explanation would be that the abundance did not change, but rather our method was more likely to detect the transcript at the 2-cell stage. This might be corrected for by unmasking the poly-adenine tail so that it could be reverse transcribed more effectively.

This apparent decrease of *TRIM28* at the 2-cell stage resulting from an absence of protein synthesis during fertilization may be a consequence of the different method of data analysis. Since cycloheximide decreased the stability of *YWHAG* mRNA, data from all cycloheximide-treated embryos were compared to the control embryos without first adjusting for mRNA levels in each sample. This method revealed a decrease in *TRIM28* abundance in cycloheximide-treated, 2-cell embryos in comparison to the control. By this approach, however, it is difficult to also determine if *TRIM28* abundance in cycloheximide-treated, 2-cell embryos is different from MII oocytes; this obstacle prevented us from properly comparing the change in *TRIM28* levels between MII oocytes and cleavage-stage embryos. Consequently, an increase of *TRIM28* from the MII oocyte to the 2-cell stage in cycloheximide-treated embryos may still occur, but we cannot be certain without a stable housekeeping gene to normalize against.

If the data analysis had no effect on expression trends, then inhibition of protein synthesis clearly decreases *TRIM28* mRNA abundance during fertilization. One mechanism for this decrease in cycloheximide-treated, IVF embryos could be due to increased mRNA degradation. Proteins, such as ELAVL1, play a role in mRNA stabilization (Brennan and Steitz, 2001). Inhibition of protein synthesis would block the translation of such essential proteins, thus causing more rapid mRNA degradation. In contrast, a similar strategy of inhibiting transcription and translation confirmed that degradation of *TET3* transcripts after the 2-cell stage is both transcription- and translation-dependent (Lee et al., 2014).

In vitro maturation had no effect on *SETDB1* abundance in the MII oocyte. *SETDB1* levels did, however, decrease throughout preimplantation development, with no difference between in vitro- and IVV embryos. This pattern of expression was similar to a previous study done in porcine embryos that reported reduction of *SETDB1* transcript quantities in 4-cell and blastocyst stage embryos in comparison to germinal-vesicle and MII oocytes (Park et al., 2010).

Oxygen partial pressue in the culture environment has also been implicated to affect early embryo development (Machaty et al., 1998). For pre-morula-stage porcine embryos, 5% O_2_ concentrations is more beneficial than 20% O_2_ (Wright, 1977). Embryos cultured in differing oxygen partial pressures showed no stage-dependent effect for *TRIM28* mRNA levels, but there was an overall effect of oxygen partial pressure on its abundance, wherein *TRIM28* expression was higher in embryos cultured in the 5% O_2_ as compared to the 20% O_2_ environment (Fig. 1).

TP53 is responsible for many cellular activities, such as apoptosis, cell cycle arrest, or cellular senescence (Vogelstein et al., 2000; Brooks and Gu, 2006). In the absence of stress, TP53 is controlled by ZNF420 (Yuan et al., 2012) and TRIM28, which recruits HDAC1 to deacetylate TP53, thereby repressing apoptosis (Tian et al., 2009). In stressed cells, ZNF420 and TRIM28 phosphorylation release the complex (Ziv et al., 2006), causing TRIM28 to relocate to the nucleolus (Yuan et al., 2012) while retaining acetylated TP53. Levels of *TP53* mRNA are almost non-existent in the MII, 2-cell, and 4-cell stages, but significantly increased in IVF, IVV, and SCNT embryos by the blastocyst stage. This profile is consistent with previous studies that report apoptosis in porcine embryos no earlier than Day 5 (Hao et al., 2003). In addition to the increase in *TP53* levels at the blastocyst stage, there was even more *TP53* mRNA in blastocyst-stage IVF embryos in comparison to IVV and SCNT embryos. This is in contrast to the expected trend based on previous data that show there is an increase in apoptosis on Day 6 in SCNT embryos compared to IVF embryos (Hao et al., 2003). One explanation for this apparent discrepancy is that the *TP53* mRNA quantity is a poor indicator of apoptosis in blastocyst-stage embryos. A second possibility—and one we hypothesize is occurring—is that the difference relates to the activity of the HDAC inhibitor, Scriptaid: SCNT embryos generated by Hao et al. (2003) were not treated with Scriptaid whereas our embryos were incubated with this inhibitor for 14–16 hr after activation. Indeed, *TP53* abundance in Scriptaid-treated SCNT blastocysts was lower than untreated SCNT embryos, which were more similar to the higher levels in IVF blastocysts than IVV blastocysts. These data further agree with research implicating that Scriptaid-treated porcine SCNT embryos possess enhanced DNA repair (Bohrer et al., 2013).

The experiments on SCNT embryos derived from HTC and ETC donor cell lines also revealed interesting results. Only 1–2% of porcine SCNT embryos transferred to a surrogate develop to term (Lai and Prather, 2003). Mouse embryos with maternal-deleted *Trim28* experience retarded growth after implantation, and ultimately resorption or fetal death (Messerschmidt et al., 2012). Blastocyst formation of maternal-deleted *Trim28* embryos was not affected, however, suggesting that blastocyst development does not correlate with *Trim28* abundance. Originally, we hypothesized that *TRIM28* mRNA abundance at the 4-cell and blastocyst stage is not adequately reprogrammed in SCNT porcine embryos derived from HTC cell lines compared to embryos from ETC cell lines. In this study, SCNT embryos from both cell lines behaved more like IVV embryos. This was true for both *TRIM28* and *TP53* expression. SCNT embryos derived from both cell lines had a significantly less *TRIM28* mRNA compared to IVF embryos at the 2-cell, 4-cell, and blastocyst stages, with no difference between SCNT and IVV embryos at any of these stages. Furthermore, SCNT embryos had the same developmental profile as IVV embryos for *TRIM28* expression, with a significant decrease from the MII oocyte throughout development. These similarities in *TRIM28* and *TP53* gene expression between SCNT embryos and IVV embryos occurred despite SCNT embryos being cultured in an identical environment as IVF embryos, originating from the same quality oocytes as those fertilized, and maturing in corresponding conditions as other in vitro-derived embryos. The similarity between IVV and SCNT blastocysts is consistent with other observations in our laboratory (Whitworth et al., unpublished).

Knockdown of *TRIM28* in the porcine oocyte had no effect on blastocyst rate, which corresponds to mouse knockout studies (Messerschmidt et al., 2012). We originally hypothesized that the increase of *TRIM28* in the IVF embryo is related to the increase in *TP53* transcripts, wherein the increase in *TP53* was a response to DNA damage inflicted by culture conditions, while *TRIM28* increased to protect the embryo from an increased incidence of TP53-mediated apoptosis. In contrast to this prediction, reducing TRIM28 in the IVF embryo did not affect *TP53* levels, suggesting that elevation in *TRIM28* mRNA is not directly related to changes in *TP53* abundance. Another possibility is that *TRIM28* was not knocked down sufficiently to see an effect on *TP53* levels. Further knockdown studies could help elucidate why *TRIM28* is abnormally expressed in IVF embryos.

Overall, this study revealed that abundance of *TRIM28*, *SETDB1*, and *TP53* mRNA is dynamically regulated during porcine early embryogenesis, and that *TRIM28* and *TP53* levels are abnormal in preimplantation embryos produced by IVF in comparison to IVV and SCNT embryos. Despite identical culture conditions, Scriptaid-treated SCNT embryos were more similar to IVV embryos than to IVF embryos. Future analysis of epigenetic marks and protein analysis could help elucidate why *TRIM28* and *TP53* expression is altered, and will aid in advancing the development of porcine preimplantation culture conditions and survival of in vitro-derived embryos.

## MATERIALS AND METHODS

### In Vitro Oocyte Collection

Oocytes for in vitro maturation were collected by aspirating antral follicles from pre-pubertal gilts. Ovaries were obtained from a slaughterhouse in Milan, MO, placed in Nalgene bottles, and transported at room temperature in an insulated container to Columbia, MO. Follicles 3–6 mm in diameter were aspirated with an 18-gauge needle connected to a 10-ml syringe. Follicular fluid was stored in 50-ml conical tubes until a pellet settled at the bottom; then the follicular fluid was drained off. The pellet was washed three times with the buffered culture medium PVA-TL HEPES (114.01 mM NaCl, 3.2 mM KCl, 2 mM NaHCO_3_, 340.06 µM NaH_2_PO_4_, 1.868 ml sodium DL-lactate solution (60%), 426.77 µM HEPES, 2 mM CaCl_2_·2H_2_O, 1.07 µM polyvinyl alcohol (PVA), 12 mM sorbitol, and 249.83 µM pyruvate). The pellet and PVA-TL-HEPES were divided among multiple Petri dishes, and cumulus-oocyte complexes (COCs) were selected for in vitro maturation based on morphology: Complexes were selected if they had multiple layers of cumulus cells, evenly distributed cytoplasm, and properly colored cumulus cells. Fifty to sixty COCs were placed in each well of a 4-well plate in 500 µl of oil-covered maturation medium for 40–44 hr in high O_2_ (5% CO_2_ in a water saturated air atmosphere).

### In Vitro Fertilization

After 40–44 hr of maturation, COCs were denuded by vortexing for 4 min at a medium speed in denuding medium (5.99 M mannitol, 3.01 µM bovine serum albumin (BSA), 0.030 g hyaluronidase, and 5 ml PVA-treated TL-HEPES). MII oocytes were selected and washed in MTBM (113.1 mM NaCl, 3.0 mM KCl, 7.5 mM CaCl_2_·2H_2_O, 20 mM Tris, 11 mM glucose, 5 mM sodium pyruvate, 0.05 mg/ml Gentamicin, 0.664 mM caffeine, and 2 mM BSA), then groups of 30–40 oocytes were placed in mineral-oil covered 50-µl drops of MTBM. Selected MII oocytes had evenly distributed cytoplasm, intact and round zona pellucidae, and the presence of a single polar body in the perivitelline space.

Thawed boar sperm was washed, diluted, and pipetted into the drops of MTBM with the MII oocytes. After 4–6 hr of incubation with sperm, the oocytes were washed in porcine zygote medium (PZM)_3_-MU1 medium (Bauer et al., 2011) and placed in 4-well plates containing oil-covered PZM_3_-MU1 and cultured in 20% O_2_ conditions.

### Culture In Vitro

Approximately 24 hr after fertilization, all embryos were moved from an environment of 20% O_2_ to a 5% O_2_ environment. A control well of embryos was cultured until Day 6, and blastocyst rates were monitored to ensure proper embryo development.

### Alpha-Amanitin Treatment

Alpha-amanitin was dissolved in PZM_3_-MU1stock, and added to the PZM_3_-MU1 culture medium to a final concentration of 22 µM. Control embryos were developed in culture medium without α-amanitin. Embryos from both treatments were cultured until Day 6 to confirm normal blastocyst development in the control group and successful inhibition of transcription in the α-amanitin treated embryos. Proper inhibition was determined by embryo development arresting at the 4- or 8-cell stage in the α-amanitin treated embryos (Schoenbeck et al., 1992).

### Cordycepin Treatment

Cordycepin, 3′-deoxyadenosine, was dissolved in RNase-free water and added to PZM_3_-MU1 culture medium to a final concentration of 40 µM. Control embryos were simultaneously placed in culture medium with no cordycepin. Embryos from both treatments were cultured until Day 6 to confirm normal blastocyst development in the control group. Proper function of the inhibitor was determined by maturing COCs in maturation medium containing cordycepin; this results in a failure of cumulus cell expansion (unpublished data).

### Cycloheximide Treatment

Cycloheximide (4-[(2*R*)-2-[(1*S*, 3*S*, 5*S*)-3, 5-dimethyl-2-oxocyclohexyl]-2-hydroxyethyl piperidine-2, 6-dione) was dissolved in ethanol and added to PZM_3_-MU1 culture medium to a final concentration of 35 µM. Control embryos were simultaneously placed in culture medium without cycloheximide. Embryos from both treatments were cultured until Day 6 to confirm normal blastocyst development in the control group. Proper function of the inhibitor was established if cycloheximide-treated embryos failed to cleave.

### Parthenogenetic Activation

MII oocytes obtained from the slaughterhouse were selected after 40–44 hr of maturation. To best simulate SCNT activation, groups of 30 oocytes were placed between two platinum wires in 0.1 mM low-calcium fusion medium (0.3 M mannitol, 0.1 mM CaCl_2_·2H_2_O, 0.1 mM MgCl_2_·6H_2_O, and 0.5 mM HEPES; pH 7.0–7.4). The platinum wires were attached to a BTX Cell Manipulator 200, and oocytes received a pulse of 1.20 kV/cm for 30 µsec. Oocytes were chemically activated by incubation at 37°C in 200 µM thimerosal for 10 min. After incubation in thimerosal, oocytes were transferred to a dish of 2 ml PVA-TL HEPES, washed, and incubated at 37°C in 8 mM dithiothreitol for 30 min. Oocytes were then washed in a dish of 2 ml PVA-TL HEPES, and transferred into a 500-µl mineral-oil covered well of 500 nM Scriptaid in PZM_3_-MU1 for 14–16 hr. After the Scriptaid treatment, embryos were washed in PZM_3_-MU1, transferred to a covered 500-µl oil-covered well of PZM_3_-MU1 medium, and cultured in 20% O_2_. Approximately 24 hr after activation, all embryos were moved from an environment of 20% to 5% O_2_. Samples past the 2-cell stage were cultured and collected from the 5% O_2_ environment.

### Somatic Cell Culture

Cryopreserved somatic-cell lines were removed from liquid nitrogen tanks, and thawed by agitating the sample tube in a 37°C water bath. Both sets of donor cells were derived from Yorkshire-by-Landrace cross fetuses. HTC donor cells were defined as “hard-to-clone” based on a previously characterized 29% pregnancy rate from embryos transferred and over 30% mortality rate of fetuses born. ETC donor cells were defined as “easy-to-clone” based on a previous, greater-than-50% pregnancy rate with healthy fetuses. Once cells were thawed, they were centrifuged in a 15-ml conical tube in 10 ml of cell culture medium (DMEM with 15% fetal bovine serum and Gentamicin (0.05 mg/ml)) for 5 min at 500*g*. The pellet was resuspended by careful pipetting in 1 ml of cell culture medium. Resuspended cells were transferred to two wells of a 4-well plate (Nunc Cell Culture dishes, Thermo Fisher Scientific, St. Louis, MO), and cultured at 37°C in 20% O_2_. After 24–48 hr of cell culture, the supernatant was removed and wells were washed with 500 µl phosphate-buffered saline (PBS). Wells were then incubated with 200 µl 0.05% trypsin for 2 min at 37°C. The trypsin activity was then stopped with cell culture medium, and the cells were centrifuged in a 15-ml conical tube in 10 ml of cell culture medium for 5 min at 500*g*. Supernatant was removed, and the cell pellet was resuspended in manipulation medium for single-cell isolation.

### Somatic-Cell Nuclear Transfer

MII oocytes with an extruded polar body were selected and placed in drops of manipulation medium (Lai and Prather, 2003) with 14 µM cytochalasin B covered with mineral oil. MII oocytes were then positioned with a holding pipette, and the injection pipette was used to penetrate the zona pellucida and to remove the polar body and surrounding cytoplasm. Enucleated oocytes were then injected, inserting one donor cell (see section above) in the perivitelline space. Injected oocytes were fused and activated as described in the “Parthenogenetic Activation” section above, in groups of 20–25.

### In Vitro Sample Collection

The following samples were collected from 20% O_2_: MII (40–43 hr), 2-cell (23–25 hr), 4-cell (30–35 hr), morula (125 hr), and blastocyst (144 hr). Samples from 5% O_2_ culture were collected at the following stages: 4-cell, morula, and blastocyst. Pools of 10–20 oocytes/embryos were collected, and the zona pellucidae were removed using a low pH saline (1.77 pH) solution. Samples were washed in 2 ml of diethylpyrocarbonate (DEPC)-treated PVA-PBS, snap frozen, and stored at −80°C. Three to four biological samples of each stage were collected.

### In Vivo Sample Collection

The oviduct of a Large White Breed sow showing standing estrus was flushed to obtain in vivo-matured MII oocytes. For 2-cell (Day 2), 4-cell (Day 2), morula (Day 4), and blastocyst (Day 5) embryos, a large white breed sow was bred at standing estrus (Day 0). Flushes were performed by either sacrificing the sow or performing survival surgery, and flushing the oviducts (Day 2) or uterine horns (Day 4 or 5) with PVA TL-HEPES. The embryos were collected and processed as mentioned above, in the “In Vitro Sample Collection” section.

### RNA Isolation and cDNA Synthesis

RNA isolation was performed from each sample using a Dynabeads^R^ mRNA Direct™ Micro-Kit (Invitrogen, Grand Island, NY). RNA extraction from a total of 3–4 biological replicates was completed for each developmental stage measured. Each replicate yielded mRNA that was then diluted in 12 µl of ultra-pure water. Due to limited numbers of IVV embryos, cDNA was amplified for selected experiments. All developmental stages for IVV, IVF, SCNT, and parthenogenetic embryos were amplified, as well as oocytes and embryos that were treated with inhibitors after fertilization. Microinjected oocytes and embryos and samples treated with inhibitors during fertilization and culture were *not* amplified.

To obtain amplified cDNA, 5 µl of the mRNA sample was used to synthesize first- and second-strand cDNA with the WT-Ovation™ Pico RNA Amplification System (NuGEN Technologies, Inc., San Carlos, CA). Amplified cDNA samples were purified by Micro Bio-Spin 30 Columns in RNase-Free Tris (Bio-Rad, Hercules, CA), and stored at −80°C. For the purpose of qPCR, an aliquot of purified amplified cDNA template was diluted to 5 ng/µl in ultra-pure water. Unamplified cDNA was synthesized using the entire 12 µl of diluted mRNA for the SuperScript® VILO™ cDNA Synthesis Kit (Invitrogen). Note that unamplified samples were used to generate the data for Figures 3 and 7.

### Quantitative Reverse-Transcriptase PCR (qPCR)

For characterization of *TRIM28*, *SETDB1*, and *TP53*, master mixes were assembled that contained: 12.5 µl 2X SYBR green mix (Bio-Rad), 10.5 µl purified water, 1 µl of 5 ng amplified cDNA template, and 0.5 µl of 20 µM forward and reverse primers designed for the specific gene of interest (Integrated DNA Technology, Coralville, IA) (Table2). An additional master mix containing identical amounts of SYBR green (Bio-Rad), purified water, cDNA template, and forward and reverse primers for *YWHAG* (Table2) was used.

**Table 2:** Primers and siRNA Sequences

	Primer orientation	Primer sequence
qPCR		
*TP53*	Forward	5′-GGAACAGCTTTGAGGTGCGTGTTT
	Reverse	5′-ATACTCGCCATCCAGTGGCTTCTT
*SETDB1*	Forward	5′-TTGGCAAAGTACTCATCACCCA
	Reverse	5′-TTGGATGACATTGCCAAAGGCT
*TRIM28*	Forward	5′-GATCATGAAGGAGCTGAACAAGCG
	Reverse	5′-TGGATCTTAGTCATGGTCCAGTGC
*YWHAG*	Forward	5′-TCCATCACTGAGGAAAACTGCTAA
	Reverse	5′-TTTTTCCAACTCCGTGTTTCTCTA
Cloning		
*TRIM28*-5′	Forward	5′-CGTGTGAATGGCGGCTTCGG
	Reverse	5′-GCTGGCTGCTGCCAGAGGTC
*TRIM28*-3′	Forward	5′-GTTGCAGAACACCAAG
	Reverse	5′-TTATTAAATCCACAGAAGTAAAAACC
*siRNA*		
*TRIM28*_A1		5′-CGAGGGACGGUGAGCGCACGGUAUA
*TRIM28*_A2		5′-UAUACCGUGCGCUCACCGUCCCUCG
*TRIM28*_B1		5′-UCAGCGGUGAGAUCCAAGUCCAGGC
*TRIM28*_B2		5′-GCCUGGACUUGGAUCUCACCGCUGA
*TRIM28*_C1		5′-ACCAAAGGCCUCGUUCAUGCGAGUC
*TRIM28*_C2		5′-GACUCGCAUGAACGAGGCCUUUGGU
*TRIM28*_control_1		5′-ACCAGAGGAACUCCCUUGCGUAGUC
*TRIM28*_control_2		5′-GACUACGCAAGGGAGUUCCUCUGGU

For each cDNA template, triplicate reactions were performed for the gene of interest and duplicates for *YWHAG*. No-template control reactions were also performed to determine if any genomic DNA contamination was present. qPCR was performed on a Bio-Rad MyiQ Single-Color Real-Time PCR Detection System (Bio-Rad, Hercules, CA). Each qPCR plate completed a three-step amplification protocol of 95°C for 3 min, 40 cycles of 95°C for 10 sec, 60°C for 30 sec, and 72°C for 30 sec, followed by a melting-curve analysis at 95°C for 1 min, 55°C for 1 min, and 31 cycles of 55°C for 10 sec. The results of the qPCR for the gene of interest were normalized to *YWHAG* values, except for cycloheximide-treated embryos. Embryos treated with cycloheximide were normalized to the control counterpart for that biological replicate. Each figure represents qPCR data obtained by corresponding biological replicates performed on an individual plate for the gene of interest and *YWHAG*.

### Cloning PCR Fragments

To obtain the porcine coding sequence for *TRIM28*, non-amplified cDNA from Day-7 blastocysts was used. To overcome the GC-rich 5′-end, the sequence was cloned in two separate pieces. Primers were designed for both the 5′- and 3′-end (Table2) by using porcine expressed-sequence tags (ESTs) that aligned to human *TRIM28* in a nucleotide BLAST (NCBI). PCR for the 5′-end was performed by using a master mix containing: 0.25 µl Q5 High-Fidelity Polymerase (New England BioLabs, Ipswich, MA), 5 µl 5X Q5 Reaction Buffer, 0.5 µl 10 nM dNTPs, 0.625 µl each 20 µM *TRIM28-5*′ cloning primer, 0.5 µl template DNA, 5 µl 5X Q5 GC Enhancer, and 12.5 µl nuclease-free water. PCR was then performed on the 5′-end samples by the following thermocycling conditions: 98°C for 30 sec, 35 cycles of 98°C for 10 sec, 72°C for 30 sec, and 72°C for 50 sec, followed by 72°C for 2 min. PCR for the 3′-end was performed by using a master mix containing: 0.25 µl Q5 High-Fidelity Polymerase (New England BioLabs), 5 µl 5X Q5 Reaction Buffer, 0.5 µl 10 nM dNTPs, 0.625 µl 20 µM each *TRIM28*-3′ cloning primer, 0.5 µl template DNA, and 17.5 µl nuclease-free water. Thermocycling conditions for the 3′-end PCR was as follows: 98°C for 30 sec, 35 cycles of 98°C for 10 sec, 64°C for 30 sec, and 72°C for 50 sec, followed by 72°C for 2 min. PCR products (6 µl) were then separated by a 0.8% agarose gel electrophoresis, and bands at approximately 1.5–2 kb were considered positive for *TRIM28* amplification.

Positive PCR products were purified (Qiagen, Valencia, CA) and cloned into a TOPO TA vector (Invitrogen). The following TOPO reaction was incubated at room temperature for an hour: 4 µl of fresh PCR product, 1 µl of salt solution, and 1 µl TOPO vector. Transformation of the TOPO reaction was performed precisely by the One Shot Chemical Transformation Protocol, and the TOP10 Competent Cells were plated at varying concentrations onto pre-warmed LB Agar plates containing 50 mg/ml kanamycin, then incubated at 37°C for 15–16 hr. Colonies were selected and placed into 3 ml of LB broth containing 50 mg/ml kanamycin, and horizontally shaken for 15–16 hr. Broth containing bacteria were processed by PureLink Quick Plasmid Miniprep Kits (Invitrogen) to obtain purified plasmid DNA.

To confirm correct size of the DNA inserted into the plasmid, a restriction enzyme digestion was performed with EcoRI (Invitrogen). The restriction digest contents containing 5 µl plasmid DNA, 2 µl 10× buffer, 12 µl sterile water, and 1 µl EcoRI enzyme were incubated at 37°C for an hour. Contents of the restriction enzyme digestion were run on a 0.8% agarose gel, and samples demonstrating the proper band size were used to identify the proper plasmid DNA to be sequenced. Sequencing was done with M13 primers at the DNA Core (University of Missouri-Columbia), and assembled with a DNA concentration between 1,200 and 1,800 ng.

### siRNA Design

*TRIM28* Stealth RNAi™ siRNA (Life Technologies, Grand Island, NY) was designed by the BLOCK-iT™ RNAi Designer (http://rnaidesigner.invitrogen.com/rnaiexpress/) by using porcine EST sequences that aligned to the human *TRIM28* sequence in a NCBI. Three siRNAs were designed to target three different regions: the 5′-end, the open reading frame, and the 3′-end (Table2). Control siRNA was designed by randomly selecting a *TRIM28* siRNA, and scrambling the middle sequence.

### siRNA Microinjections

Duplex siRNAs were resuspended in RNase-free water at a stock concentration of 200 µM. Equal concentrations of *TRIM28*_A, *TRIM28*_B, and *TRIM28*_C were diluted into a cocktail siRNA with a final concentration of 1 µM siRNA with 50 ng/µl GFP mRNA. The control group contained *TRIM28*_control siRNA at a final concentration of 1 µM siRNA with 50 ng/µl GFP mRNA. Oocytes were denuded at 30–33 hr of maturation, and injected with either the 1 µM siRNA or Control siRNA. Microinjections were performed with a FemtoJet (Eppendorf, Hauppauge, NY) with a constant pressure. Microinjected oocytes were placed back in maturation medium. At 40–44 hr of maturation, MII oocytes were selected and placed under ultraviolet light to determine fluorescence. GFP-positive MII oocytes were fertilized as previously mentioned in “In Vitro Fertilization.” Day-6 blastocysts were collected for qPCR.

### Statistical Analysis

All mRNA abundance data (except the cycloheximide-treated) were normalized to *YWHAG*, and uploaded into Statistical Analysis System software. For all data, the PROC UNIVARIATE was run first to determine if the data was normally distributed. If the data were not normally distributed, they were log2 transformed before further analysis. A PROC GLM was then run for each experiment. For each gene of interest, two models were run. First, a model that looked at statistical difference within a treatment across stages of development. Then, a model that analyzed differences between treatments within a single stage of development was constructed. Since all the developmental stages and treatments could not fit on a single plate, replicate samples were run on one plate with all the developmental stages for a specific gene. Next the samples were rerun with the separate treatments on a single qPCR plate.
